# Influenza Vaccination Coverage and Influencing Factors in Type 2 Diabetes in Mainland China: A Systematic Review and Meta-Analysis

**DOI:** 10.3390/vaccines12111259

**Published:** 2024-11-06

**Authors:** Cheng Yang, Shijun Liu, Jue Xu, Wen Fu, Xin Qiu, Caixia Jiang

**Affiliations:** Department of NCDs Control and Prevention, Hangzhou Center for Disease Control and Prevention (Hangzhou Health Supervision Institution), Hangzhou 310021, China; yc_hzcdc@163.com (C.Y.); shijun9170@sina.com (S.L.); xu.jue@outlook.com (J.X.);

**Keywords:** T2DM, influenza vaccination, China, systematic review, meta-analysis

## Abstract

Background: Influenza has many harmful effects on people with type 2 diabetes mellitus (T2DM), such as hyperglycemia and increasing incidence of cardiovascular and cerebrovascular diseases. Epidemiological evidence shows that influenza vaccinations can effectively prevent deterioration in T2DM patients. At present, there is a lack of nationwide studies on the vaccination status of influenza vaccines for patients with certain chronic diseases. This study aimed to evaluate the influenza vaccination status of T2DM patients in mainland China and the factors affecting their influenza vaccination. Methods: Data were sourced from PubMed, Embase, Web of Science, the China Biology Medicine Disc (CBMdisc), the China National Knowledge Infrastructure (CNKI), and the Wanfang Database. The keywords used in the literature search included “diabetes”, “diabetes mellitus”, “DM”, “diabetic”, “T2DM”, “influenza vaccine”, “flu vaccine”, “China”, and “Chinese”. A total of 249 articles were retrieved through the searches; 7 articles met the inclusion criteria. The fixed-effects model was used when heterogeneity was low and a random-effects model was used when the heterogeneity was high. Results: The influenza vaccination coverage rate was 1.46% in diabetic patients and 9.99% in elderly diabetic patients. The influenza vaccination rate of type 2 diabetes patients with a high education level is higher than that of patients with a low education level. (OR: 1.462 [1.123, 1.903]). Meanwhile, gender (OR: 1.076, 95%CI: 0.893–1.295), marriage (OR: 1.283; 95%CI: 0.931–1.766), and occupation (OR: 1.049; 95% CI: 4.422–2.606) have no significant impact on influenza vaccination in patients with type 2 diabetes. Conclusions: This study found that the coverage of influenza vaccination in patients with T2DM in Chinese mainland was low, and there were few relevant research articles. In China’s mainland areas, education background is an important factor affecting the influenza vaccination of T2DM patients. China should continue to improve the influenza vaccination rate of patients with type 2 diabetes.

## 1. Introduction

According to the “Global Diabetes Map” released by the International Diabetes Federation (IDF), as of 2021, there were about 141 million people with diabetes aged 20–79 in China, with an incidence rate of 12.8%, and it was estimated that 51.7% of diabetes patients had not been diagnosed [1]. Such a large population means diabetes is a heavy burden on China’s healthcare system. Complications and comorbidities of diabetes not only seriously affect the health and quality of life of patients, but also may lead to premature death [2].

Infection with the influenza virus typically causes a self-limited illness with high fever, myalgias, and malaise [3,4], and may also accelerate acute thrombotic vascular events, particularly in patients with ischemic heart disease and cerebrovascular disease [5,6,7]. People with diabetes are more vulnerable to the flu than healthy people, and influenza can progress to more serious consequences, such as pneumonia, myocardial infarction, stroke, and an increased risk of premature death [8,9,10,11,12]. Therefore, many countries recommend that people with T2DM, especially the elderly, need to be prioritized for influenza vaccination, but reality has fallen short of expectations.

Many existing studies have shown the protective effect of influenza vaccination in people with T2DM. In a large population-based study, influenza vaccination in people with diabetes was associated with reductions in rates of hospital admission for acute cardiovascular and respiratory diseases, and in all-cause mortality across seven influenza seasons [13]. A cohort study in China demonstrated that the protective effect of vaccination can reduce the incidence of chronic kidney disease (CKD) and end-stage renal disease (ESRD) in patients with T2DM [14]. It could be seen that the influenza vaccine can offer comprehensive protection for individuals with T2DM.

There has been extensive exploration on the factors affecting influenza vaccine coverage worldwide. Influencing factors include individual factors such as gender, age, occupation, income, and educational attainment, as well as social factors such as urban economic development and social support. The impact of individual factors on influenza vaccine coverage in patients with T2DM is more complex, and the same individual factor can show different effects in different countries.

In mainland China, limited monitoring and research on influenza vaccination among the T2DM subpopulation hinder the ability of scientists to assess vaccination rates. The main purpose of this study was to understand the current situation of influenza vaccination in patients with T2DM, and to explore the factors affecting influenza vaccination in the context of a Chinese cultural background, so as to provide a scientific basis for more targeted influenza vaccination in patients with T2DM in the future.

## 2. Materials and Methods

This study followed the Preferred Reporting Items for Systematic Reviews and Meta-analyses (PRISMA) guidelines [15]. This study has been registered with the International Prospective Register of Systematic Reviews (PROSPERO; CRD42024569815). Foreign literature was retrieved from PubMed, Embase, and Web of Science. The CBMdisc, CNKI, and Wanfang databases provided Chinese literature. The search period was from January 2010 to December 2023. Keywords, among others, included “diabetes”, “diabetes mellitus”, “DM”, “diabetic”, “T2DM”, “influenza vaccine”, “flu vaccine”, “China”, and “Chinese”. The complete search strategy is attached as supplemental online material. We based our age classification of elderly patients with T2DM on the definitions employed in each study.

Inclusion criteria: (1) patients with T2DM; (2) research in mainland China; (3) observational studies; (4) study results should include influenza vaccination coverage in T2DM patients or factors affecting vaccination. The study was excluded if one of the following held: (1) there was no full text; (2) duplicate reports; (3) lack of representativeness of research subjects; (4) the timing of influenza vaccine was uncertain; (5) Newcastle–Ottawa scale (NOS) score < 6 or Agency for Healthcare Research and Quality (AHRQ) score < 7; (6) the article was a review, case report, or conference paper.

Microsoft Excel (Version 2409 Build 16.0.18025.20160) was used to capture all information required for the analysis, including the first author, publication date, study design, study time, study location, number of subjects, demographic characteristics of subjects (including age, sex, education, marital status, etc.), number of influenza vaccinations, influenza vaccination coverage, etc.

The cohort studies were evaluated using the NOS [16], and the cross-sectional studies were evaluated concerning the rating scale developed by the AHRQ [17].

The coverage rate of the influenza vaccine in T2DM patients, odds ratio (OR) of each influencing factor, and 95% confidence intervals (CIs) were used as effect size indicators.

Logarithmic conversion was used when merging influenza vaccination rates. The inconsistency index (I2) was used to assess heterogeneity with values of 25%, 50%, and 75% considered low, moderate, and high, respectively [18]. When I2 > 50%, this study used a random-effects model; when I2 ≤ 50%, this study used a fixed-effects model. Subgroup analysis was conducted to investigate the impact of time on influenza vaccination in patients with T2DM.

A publication bias test and sensitivity analysis were not performed in this study for the following reasons: (1) the single group rates were descriptive results, and there were no “positive” results or statistically significant results, so a publication bias test and sensitivity analysis were not required; (2) the number of studies included in the pooled analysis was limited, making it unsuitable to conduct sensitivity analysis and publication bias testing.

The study lasted from January to July 2024. The meta-analysis was performed using the metafor package in RStudio software (version 2024.04.1+748), using R language version 4.4.0 [19].

## 3. Results

### 3.1. Study Selection and Characteristics

**Figure 1:**
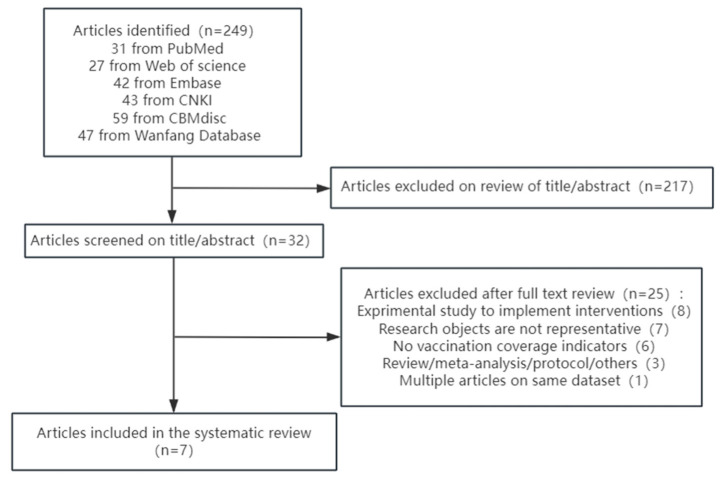
Flow diagram of study selection process.

The study selection and characteristics are shown in Figure 1. As of 31 December 2023, a total of 249 articles were retrieved through subject-term and free-term searches, 32 of which were evaluated using the full text after selecting titles and/or abstracts. We excluded some experimental studies that did not provide an influenza vaccine coverage rate and studies that met the exclusion criteria, such as a lack of representative subjects. In the end, seven articles met the inclusion criteria.

The main features of the seven articles included in this study are shown in Table 1. A total of 1,044,309 people with T2DM were included in the seven studies, of which 59,596 were vaccinated with the influenza vaccine. Most of the research focused on the period of 2016–2018, with cross-sectional analysis being the predominant design. The majority of studies had an influenza vaccine coverage of less than 10%, and only the study in Taizhou demonstrated a rate of more than 10% (28.7%) [15]. It can be intuitively seen that the coverage of the influenza vaccine after 2016 was higher than before 2016. Among the seven articles included in this study, five reported on the influenza vaccination coverage rate in elderly patients with T2DM [20,21,22,23,24] and three explored the influencing factors of the influenza vaccination coverage rate in patients with T2DM [20,21,23].

### 3.2. Influenza Vaccine Coverage Rate and Subgroup Analysis in Patients with T2DM

#### 3.2.1. Overall Patients with T2DM

**Figure 2:**
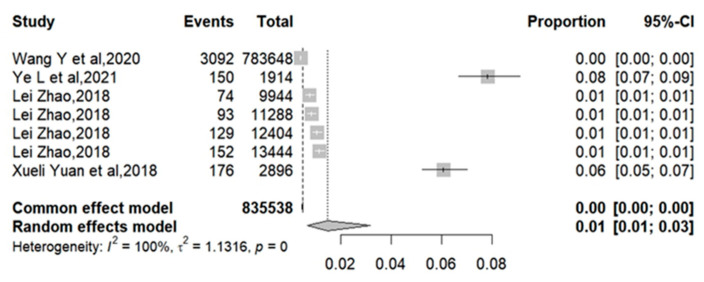
The vaccination coverage rate in overall patients with T2DM [20,23,25,26].

**Figure 3:**
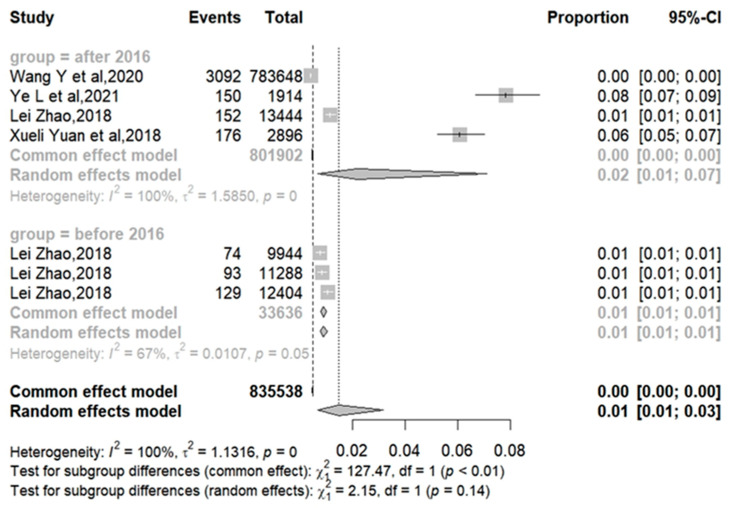
The vaccination coverage rate in T2DM patients during the study period [20,23,25,26].

The influenza vaccination rates for T2DM were reported in four articles as 0.39%, 7.84%, 0.74%, 0.82%, 1.04%, 1.13%, and 6.08%, respectively [20,23,25,26]. The meta-analysis showed that the pooled influenza vaccination coverage rate was 1.46% (95% CI: 0.67%–3.17%) with high heterogeneity (I2 = 99.8%) (Figure 2). Subgroup analysis based on the study period reported that the heterogeneity of influenza vaccination rates among groups before 2016 was relatively low (I2 = 67.2%), with a pooled coverage rate of 0.87% (95% CI: 0.74%–1.03%). The heterogeneity of research after 2016 remained high (I2 = 99.7%), with the meta-analysis showing a pooled rate of 2.18% (95% CI: 0.64%–7.1%) (Figure 3).

#### 3.2.2. Elderly T2DM Patients

**Figure 4:**
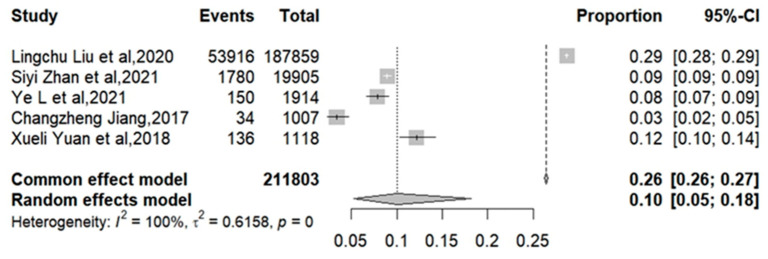
The influenza vaccination coverage rate in elderly patients with T2DM [20,21,22,23,24].

The meta-analysis results of influenza vaccination coverage in elderly patients with T2DM are shown in Figure 4. The five studies included were all conducted after 2016. Four of the five articles reported an influenza vaccination rate of more than 5%, with the highest coverage rate being 28.7% in a Taizhou’s study [22]. The pooled influenza vaccine coverage rate in elderly T2DM patients of the random-effects model was 9.99% (95%CI: 5.26%–18.16%), with high heterogeneity (I2 = 99.9%).

### 3.3. Association of Gender, Marital Status, Occupation, and Education Attainment with Influenza Vaccination in T2DM

**Figure 5:**
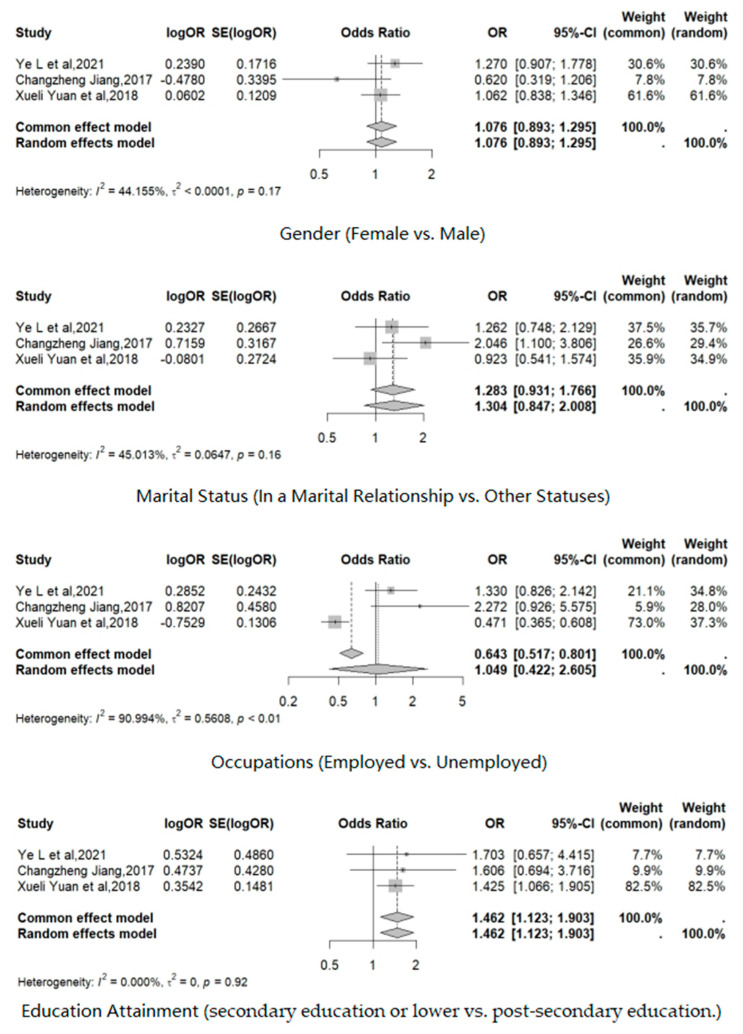
Association of gender, marital status, occupation, and education attainment with influenza vaccination in T2DM patients [20,21,23].

The following three included articles described the vaccination coverage rate of T2DM patients with different demographic characteristics (Figure 5). We selected the following factors (gender, marital status, occupation, and education attainment), which were analyzed in these articles for this meta-analysis. Gender was divided into male and female. Marital status was divided into being in a marital relationship and others. Occupation was classified into two categories: employed and unemployed. Education attainment was divided into secondary education or lower and post-secondary education.

No significant differences were found between different genders in the included studies; the pooled OR by the fixed-effects model was 1.076, 95% CI: 0.893–1.295 (I2 = 44.2%, 95% CI: 0.0%–83.3%). Research in Ningbo suggested that the influenza vaccination coverage rate in females was higher than in males (OR 0.62, 95% CI: 0.316–1.196) [21], and the other two papers indicated the opposite (OR 1.27, 95%CI: 0.908–1.779; OR 1.062, 95%CI: 0.838–1.346) [20,23].

A study conducted in Jiangdong found a significant association between marital status and influenza vaccination among T2DM patients (OR 2.046, 95%CI: 1.1–3.806), indicating that married T2DM patients were more likely to receive the influenza vaccine [21]. The other two articles did not show significant differences. The pooled OR by the fixed-effects model was 1.283 (95%CI: 0.931–1.766; I2 = 46.4%).

The impact of the employment of T2DM patients on their influenza vaccination behavior was different in different regions. The research in Shenzhen indicated that the influenza vaccination rate among unemployed patients with T2DM was significantly lower than that of their employed counterparts (OR 0.471, 95%CI: 0.365–0.609) [23]. However, the other two studies suggested that the employment status might not be associated with influenza vaccination. Nonetheless, the numerical data showed that vaccination rates were higher among employed individuals with T2DM. Consequently, these three studies exhibited considerable heterogeneity (I2 = 91.0%); this meta-analysis did not observe a significant effect of occupational status on influenza vaccination (OR 1.049, 95%CI: 0.4224–2.6055).

In the three selected articles, the influenza vaccination rate of T2DM patients having received a post-secondary education was significantly higher than that of their counterparts with a secondary education or lower. The study conducted in Shenzhen highlighted that the differences in vaccination rates across various educational levels were statistically significant (OR 1.425, 95%CI: 1.066–1.905) [23]. Furthermore, the three included articles exhibited a high degree of homogeneity regarding this factor. The fixed-effects model revealed a pooled odds ratio of 1.462 (95%CI: 1.123–1.903), indicating that T2DM patients with higher education levels were more likely to receive influenza vaccinations.

## 4. Discussion

It was required that the study populations included were representative in the selection of studies. Certain studies were found to have excluded type 2 diabetes patients with specific characteristics, such as those without complications or comorbidities. These characteristics may have influenced their influenza vaccination status. We also required that the articles specify the timing of vaccination for T2DM patients. We determined that vaccination should have occurred within one year prior to the initiation of the investigation. Furthermore, the term “time” refers to the influenza vaccination coverage within a specific year, rather than the publication date of the article. All included studies were assessed using the NOS or AHRQ criteria. The scores of these articles were found to be relatively balanced, reflecting their similarities in terms of study populations and methodologies. The articles included in this study exhibited several notable characteristics: (1) the literature was updated infrequently; (2) the number of relevant studies was limited; and (3) the study locations were predominantly in more developed cities.

Descriptive studies on influenza vaccination coverage among T2DM patients in mainland China have progressed slowly. Most of the existing research was conducted between 2016 and 2019. Some investigations were focused on assessing the effectiveness and protective role of vaccinations, while others explored the factors that influenced changes in vaccination intention. South Korea had conducted comprehensive studies analyzing trends and influencing factors related to influenza vaccination in T2DM patients from 2007 to 2019 [27]. These studies utilized nationwide data, ensuring good representativeness of the subjects. The research in China was primarily derived from economically developed cities such as Zhejiang, Shanghai, and Shenzhen. Even among the studies that were excluded, the majority originated from these cities. The Chinese government has promoted free influenza vaccinations for elderly patients with T2DM in areas where resources allowed. This policy was more readily implemented in economically developed regions like Beijing and Shanghai, while underdeveloped areas faced significant challenges. Consequently, the assessment of influenza vaccination rates among patients with type 2 diabetes in underdeveloped areas was of paramount importance. The lack of such surveys obstructed our ability to obtain a comprehensive understanding of influenza vaccination patterns and further hindered a national evaluation of policy effectiveness.

The pooled analysis revealed that the influenza vaccine rate among T2DM patients in China remained low, with most studies reporting rates below 10%. Furthermore, significant regional variations contributed to considerable heterogeneity within the meta-analysis. For context, the vaccination rate for diabetic patients over 18 years in the United States reached 61.6% in 2015 [28], while South Korea reported a rate of 59.8% in 2019 [27]. In contrast, only 6.4% of diabetic patients in Malaysia received the influenza vaccine [29]. Clearly, the flu vaccination rate for T2DM patients in China was substantially lower compared to many developed nations, and similar low rates have been observed in developing countries. This suggests that a country’s economic development level may influence its vaccination rates. For subgroup analysis, the seven studies on influenza vaccine coverage were categorized into two groups (pre-2016 and post-2016). The analysis indicated that the heterogeneity of influenza vaccination rates decreased prior to 2016, yet the heterogeneity of vaccination rates remained high subsequent to 2016. This discrepancy might be attributed to several factors: (1) the coverage rates before 2016 were derived from a single city; (2) overall influenza vaccine coverage among diabetes patients in China has been steadily increasing; and (3) the implementation of the “free influenza vaccine” policy varied significantly across cities, leading to imbalances in vaccine coverage rates in different regions after 2016.

The results suggested that the influenza vaccination coverage rate among elderly T2DM patients was higher than that of T2DM patients of all ages. This phenomenon primarily arose from the widespread recognition that elderly individuals are particularly vulnerable to influenza. Adults with T2DM were at increased risk for influenza-related morbidity and mortality, and they often exhibit an impaired immune response to the influenza vaccine, making them more susceptible to complications [30]. Furthermore, the elderly population is universally recognized as being at heightened risk for influenza-related complications, hospitalization, and mortality when compared to young, healthy adults [31]. Therefore, it is imperative that older individuals with T2DM receive adequate protection through vaccination. The Technical Guidelines for Influenza Vaccination in China (2022–2023) emphasized the necessity of prioritizing influenza vaccination for elderly patients with diabetes [32]. This further illustrated the Chinese government’s commitment to enhancing influenza vaccination coverage among elderly T2DM patients through various initiatives.

Gender, marital status, occupation, and educational attainment were identified as factors explored in relation to influenza vaccination among T2DM patients. The meta-analysis revealed high homogeneity across these influencing factors (I2_gender_, I2_occupation_, and I2_education_ attainment were all less than 50%). This indicated that, despite variations in population characteristics and economic conditions across regions, the effects of these factors on influenza vaccination among T2DM patients remain relatively stable. Notably, the analysis found differences in vaccination rates based on education attainment, with individuals holding higher educational qualifications being more likely to receive the influenza vaccine. This finding stands in stark contrast to certain international studies, which have observed that individuals with higher educational attainment often exhibit lower vaccination rates [33,34,35]. However, other research conducted in China supported the notion that T2DM patients with higher education levels are associated with significantly higher vaccination rates [36,37]. This discrepancy might stem from the limited social support and awareness among less-educated T2DM patients regarding the risks of influenza and the benefits of vaccination. Consequently, it is imperative for the government to enhance influenza vaccination initiatives targeted at T2DM patients with lower educational attainment.

No significant differences were observed in relation to gender and marital status. However, this did not imply that these factors had no influence on influenza vaccination among individuals with T2DM. Notably, one included article indicated that marriage significantly influenced influenza vaccination among T2DM patients [21]. Regarding occupational factors, the results from the study displayed considerable variability among studies. For instance, one study from Shenzhen reported that unemployed T2DM patients were more likely to receive the flu vaccine compared to their employed counterparts [23]. This finding contradicted the results of two other studies. It is reasonable to speculate that these differences may be linked to varying economic conditions or occupational demographics in different cities. Unfortunately, the available literature did not provide further evidence or discussion on this matter. Therefore, additional research is essential to investigate the relationship between occupation and influenza vaccination among T2DM patients. Some studies from other regions in China corroborated the findings of this study [38,39,40].

A French study [41] identified several factors contributing to low influenza vaccination rates among individuals with diabetes. These included ignorance about the potential adverse effects of influenza, lack of awareness regarding diabetes, concerns about the safety of influenza vaccine, and distrust arising from past experiences where the vaccine did not meet expectations. Such factors influenced the willingness and behavior of diabetics towards vaccination, regardless of the country. In China, the coverage rate for influenza vaccination remains inadequate. To address this issue, vaccination sites should implement targeted strategies to engage T2DM patients, particularly those with low vaccination rates and limited willingness to vaccinate. By adopting these measures, it is possible to enhance the influenza vaccination rate and better protect T2DM patients in our country from the severe consequences of influenza.

The COVID-19 pandemic has significantly impacted influenza vaccination efforts among individuals with diabetes. The shift in the focus of healthcare services and the reduction in patient visits contributed to a decrease in vaccination rates among this vulnerable population. Many patients prioritized COVID-19 vaccination, neglecting routine vaccinations like influenza. The current study highlights the critical role of the influenza vaccine in preventing the exacerbation of diabetes complications caused by influenza. Moving forward, targeted interventions and awareness campaigns are essential to promote influenza vaccination in diabetes management.

This study has several limitations. There was high heterogeneity across the included studies, and data on influenza vaccination coverage rates were limited. We based our age classification of elderly patients with T2DM on the definitions employed in each study, which may have varied. Thus, the findings of our study concerning the elderly population were subject to bias. Nonetheless, a meta-analysis of observational studies can yield valuable insights, especially when experimental studies are unavailable or are inappropriate for addressing a question [42]. It is important to acknowledge that the findings from this study were based on observational data, which means causality cannot be established.

## 5. Conclusions

Descriptive studies on influenza vaccination in T2DM patients in China are few and of average quality, making it impossible to accurately assess vaccination in China. Judging from the articles available, the current flu vaccine coverage among people with T2DM remains low. Education level is an important factor influencing influenza vaccination in T2DM patients in China. More high-quality studies are needed to assess the reality of influenza vaccination in T2DM patients in China in order to evaluate and improve current vaccination policies.

## Figures and Tables

**Table 1:** Characteristics of studies included in this meta-analysis.

Study	Study Period	Study Location	Study Design	Sample Source	Diabetic Patients	Patients with Diabetes Who Received Influenza Vaccine	Influenza Vaccine Coverage Rate (%)
Wang Y et al., 2020 [25]	2016–2017	Shanghai	Retrospective cohort	All diabetic patients	783,648	3092	0.39
Ye L et al., 2021 [20]	2016.8–2017.4	Ningbo	Cross-sectional	All diabetic patients	1914	150	7.84
Xueli Yuan et al., 2018 [23]	2017	Shenzhen	Cross-sectional	All diabetic patients	2896	176	6.08
Lingchu Liu et al., 2020 [22]	2018	Taizhou	Cross-sectional	Elderly diabetic patients	187,859	53,916	28.7
Siyi Zhan et al., 2021 [24]	2017.10–2018.3	Ningbo	Retrospective cohort	Elderly diabetic patients	19,905	1780	8.94
Changzheng Jiang, 2017 [21]	2014.8–2016.8	Ningbo	Cross-sectional	Elderly diabetic patients	1007	34	3.38
Lei Zhao, 2018 [26]	2013.9–2014.3	Ningbo	Cross-sectional	All diabetic patients	9944	74	0.74
	2014.9–2015.3				11,288	93	0.82
	2015.9–2016.3				12,404	129	1.04
	2016.9–2017.3				13,444	152	1.13

## Data Availability

Data are contained within the article.
